# Arbuscular Mycorrhizal Fungus *Rhizophagus irregularis* Increased Potassium Content and Expression of Genes Encoding Potassium Channels in *Lycium barbarum*

**DOI:** 10.3389/fpls.2017.00440

**Published:** 2017-03-29

**Authors:** Haoqiang Zhang, Suzhen Wei, Wentao Hu, Longmin Xiao, Ming Tang

**Affiliations:** ^1^College of Forestry, Northwest A&F UniversityYangling, China; ^2^College of Forestry and Landscape Architecture, South China Agricultural UniversityGuangzhou, China; ^3^Weihai Ocean Vocational CollegeRongcheng, China

**Keywords:** arbuscular mycorrhiza fungi, *Lycium barbarum*, drought, potassium status, potassium channels

## Abstract

Potassium in plants accounts for up to 10% dry weight, and participates in different physiological processes. Under drought stress, plant requires more potassium but potassium availability in soil solutes is lowered by decreased soil water content. Forming symbiosis with arbuscular mycorrhizal (AM) fungi not only enlarges exploration range of plant for mineral nutrients and water in soil, but also improves plant drought tolerance. However, the regulation of AM fungi on plant root potassium uptake and translocation from root to shoot was less reported. In current study, the effect of an AM fungus (*Rhizophagus irregularis*), potassium application (0, 2, and 8 mM), and drought stress (30% field capacity) on *Lycium barbarum* growth and potassium status was analyzed. Ten weeks after inoculation, *R. irregularis* colonized more than 58% roots of *L. barbarum* seedlings, and increased plant growth as well as potassium content. Potassium application increased colonization rate of *R. irregularis*, plant growth, potassium content, and decreased root/shoot ratio. Drought stress increased colonization rate of *R. irregularis* and potassium content. Expression of two putative potassium channel genes in root, *LbKT1* and *LbSKOR*, was positively correlated with potassium content in root and leaves, as well as the colonization rate of *R. irregularis*. The increased *L. barbarum* growth, potassium content and genes expression, especially under drought stress, suggested that *R. irregularis* could improve potassium uptake of *L. barbarum* root and translocation from root to shoot. Whether AM fungi could form a specific mycorrhizal pathway for plant potassium uptake deserves further studies.

## Introduction

Potassium is one of the most important mineral element in plants, which accounts for 2–10% of plants dry weight, and its concentration in cytoplasm must be maintained in the range of 100–200 mM to ensure enzymes activities (Leigh and Wyn Jones, 1984). In plants, potassium participates in multiple processes such as enzyme activation, membrane transportation, anion neutralization, and osmoregulation (Wang and Wu, 2013; Garcia and Zimmermann, 2014). Although potassium in soil is highly abundant, the majority of potassium in soil was dehydrated and coordinated to oxygen atoms. Only 0.1–1 mM potassium was available in soil solute for plants (Maathuis, 2009). The gap between high demand and low availability of potassium drove plants to develop high-affinity transport systems for potassium acquisition by root from soil (Nieves-Cordones et al., 2014), and cooperate with microorganisms (Egamberdiyeva, 2007; Garcia and Zimmermann, 2014).

Arbuscular mycorrhizal (AM) fungi, from the monophyletic phylum Glomeromycota, are ubiquitous in different ecosystems and could infect more than 80% terrestrial plants root to form mutualistic symbiosis, in which AM fungi acquire up to 20% photosynthates from plants, and supply mineral nutrients and water in return (Bago et al., 2000; Parniske, 2008; Smith and Read, 2008). For potassium, the early study of George et al. (1992) indicated that the extraradical hyphae of AM symbiosis could absorb potassium from soil, and provided the evidence that AM fungi could supply host plant with potassium. The accumulated potassium in different parts of AM fungus and mycorrhizal root section suggested that the AM fungi were capable of potassium absorption and transportation from soil to plant root (Scheloske et al., 2004; Pallon et al., 2007; Olsson et al., 2008). In plant, potassium content was highly correlated with phosphate content (Olsson et al., 2011), and played the major role as a counter-ion in polyphosphate stabilization (Orlovich and Ashford, 1993; Kikuchi et al., 2014). Considering the indispensable role of phosphate in AM symbiosis (Javot et al., 2007), potassium transportation may also played a possible role in maintaining AM symbiosis.

Drought is the seriously abiotic stress that reduces plant growth and productivity. Under drought stress, plants need potassium for stomatal regulation, osmotic adjustment, cell-membrane integrity and function. As the mobility of potassium decreased with soil water content, the gap between demand and acquirement of plant would expanded and resulted in potassium deficiency (Hu and Schmidhalter, 2005). AM fungi were reported to improve plant drought tolerance/resistance via improving water transportation, osmotic adjustment, gas exchange, and protection against oxidative damage (Ruiz-Lozano et al., 2012a; Barea et al., 2013), while extra potassium fertilization was beneficial for all these processes (Anschütz et al., 2014). Ruiz-Lozano et al. (1995) indicated that the respond of mycorrhizal plant to drought stress was closely correlated with plant potassium content. El-Mesbahi et al. (2012) found potassium supplement could improve root hydraulic properties only in mycorrhizal maize. Under salinity, which may cause physiological drought, AM fungi were reported to increase the potassium content in host plants (Porras-Soriano et al., 2009; Estrada et al., 2013). However, the regulation of AM fungi on plant genes in charge of potassium uptake by root and translocation from root to shoot was less reported.

A large number of genes encoding potassium channels were cloned, identified, and separated into three families (Shaker, TPK, and Kir-like) in plants (Wang and Wu, 2013). Within the Shaker family, genes were classified into five subfamily (AKT1 subfamily, KAT1 subfamily, AKT2 subfamily, AtKC1 subfamily, and SKOR subfamily) by sequence similarities and common functional properties. Channels are inward-rectifying from AKT1 subfamily and KAT1 subfamily, weakly inward-rectifying from AKT2 subfamily, modulator of other inward-rectifying channels from AtKC1 subfamily, and outward-rectifying from SKOR subfamily (Pilot et al., 2003; Gambale and Uozumi, 2006). In *Arabidopsis thaliana*, inward-rectifying AKT1, from the AKT1 subfamily, played a major role in root potassium uptake (Lagarde et al., 1996), while outward-rectifying SKOR, from SKOR subfamily, controlled the release of potassium into xylem sap, and contributed to the potassium translocation from root to shoot (Gaymard et al., 1998). In order to illustrate the influence of AM fungi on plant potassium uptake and translocation, the transcript of *AKT1* and *SKOR* orthologue genes were analyzed in current study.

*Lycium barbarum* L. (Solanaceae) is a perennial ligneous shrub widely raised in northwest of China, where water deficiency is the critical factor limited plant growth (Yang et al., 2011). As a drought-tolerant plant species, *L*. *barbarum* produced highly valued medical fruits (Zhao and Zeng, 1999; Gan et al., 2004; Luo et al., 2006), and formed symbiosis with AM fungi (Zhang et al., 2010). Two putative orthologue genes encoding AKT1 and SKOR were isolated from *L. barbarum*, and designated as *LbKT1* and *LbSKOR*. The objectives of current study were: (1) to evaluate the combined effect of potassium application, drought, and AM fungus on plant growth and potassium status, (2) to analyze the regulation of AM fungus on the expression of *LbKT1* and *LbSKOR* under different potassium and water conditions.

## Materials and methods

### Growth medium and plant material

Growth medium was a mixture of soil and sand (1:1, v/v). Soil used in current study was collected from the top layer of Northwest A&F University campus nursery, in Yangling City, Shaanxi province, China. Soil contained organic matter 11.85 g·kg^−1^, available nitrogen 41.25 mg·kg^−1^, available phosphorus 9.63 mg·kg^−1^ and available potassium 134.62 mg·kg^−1^. Soil pH was 7.5 (soil: water = 1.0: 2.5). Soil was ground, passed through a 2 mm sieve, and mixed with thoroughly washed river sand. Growth medium was autoclaved at 0.11 MPa and 121°C for 2 h, then placed 1 week before use.

Seeds of *L. barbarum* L. (cultivar, Ningqi No. 1) were kindly offered by Dr. Yajun Wang (Ningxia Academy of Agriculture and Forestry Sciences). Seeds were first surface sterilized with 5% sodium hypochlorite for 10 min and washed three times with sterile water, and then geminated on moist filter paper in dark at 28°C. Germinated seeds were cultivated in seedling tray (50 mL for each hole) filled with sterilized sand. Seeds were fertilized with 10 mL 1/2 Hoagland's solution (Hoagland and Arnon, 1950) every week. After 3 weeks, uniformed seedlings were selected and planted in plastic pot (13 × 11 × 9 cm) containing 1 kg growth medium. Each port was planted with two seedlings.

### AM fungal inoculation

*Rhizophagus irregularis* was provided by the Plant Nutrition and Resources Institute of Beijing Academy of Agriculture and Forestry, and propagated in pot culture with *Trifolium repens* using sterile sands prior current experiment. The result mixture consisting of spores (50 spores g^−1^), mycelium, and root fragments were used as AM fungal inoculum.

For AM fungal inoculation, 10 g inoculum was placed in plastic pot next to the uniformed *L. barbarum* seedling, while the non-mycorrhizal treatment received 10 g autoclave inoculum with microbial wash (1-μm nylon mesh) from the inoculum.

### Experimental design and growth condition

The experiment consisted of a randomized block design with three factors (2 × 2 × 3): (1) AM status, inoculated with *R. irregularis* (AM) or not (NM); (2) water status, well-watered (WW, 80% of field capacity) or drought stressed (DS, 30% of field capacity); and (3) potassium application, K0 (without KCl application), K1 (applied with 2 mmol KCl), and K2 (applied with 8 mmol KCl). Soil water content was controlled by weighing the pots every day. For potassium application, KCl solution was applied to the corresponding treatment and reached the target quantity in the fifth day to avoid an osmotic shock. There were five replicates for each treatment (12 in total), totaling 60 pots (two seedlings per pot).

The experiment was conducted in greenhouse with 12 h light per day at the temperature of 25–35°C and the relative humidity of 55–78%. Pots were arranged in a randomized complete block design. After transplantation, seedlings were fertilized with 10 mL Hoagland's solution every 10 days. Forty days post transplantation, potassium application started. Fifty days post transplantation, water treatment started and lasted for 20 days.

### Plant growth, mycorrhizal colonization, and potassium content analysis

Seventy days after transplantation, seedlings were harvested. Roots from each treatment were collected and washed with tap water to remove soil particles. Part of roots were cut into 1 cm fragments and stained with trypan blue (Phillips and Hayman, 1970). Mycorrhizal colonization was measured by the gridline intercept method (Giovannetti and Mosse, 1980).

Potassium content in leaves and roots were measured by atomic absorption spectrometry.

### RNA extraction and first-strand cDNA synthesis

RNA was extracted from frozen milled leaf and root sample using the omega E.Z.N.A™ Plant RNA Kit (Omega bio-tek, http://www.omegabiotek.com/). Quality and quantity of the RNA were assessed by measuring the A260/A280 ratio with NanoDrop 1000 spectrophotometer (Thermo-scientific, USA). The first-strand cDNA was synthesized using PrimerScript 1st Stand cDNA Synthesis Kit (Takara) from 1 μg RNA in the final volume of 15 μL.

### Isolation and computational analysis of putative genes encoding potassium channels

Based on the result of *de novo* transcriptome sequencing of *R. irregularis* colonized *L. barbarum* roots, two novel full-length cDNA sequences encoding potassium channel protein were obtained, and designated *LbKT1* and *LbSKOR*. Subsequent PCR was performed using cDNA from *L. barbarum* roots (both AM and NM treatment under WW condition) as template. Purified PCR products were transformed into the pMD18-T simple vector (Takara) for sequencing (Sunnybio, www.sunnybio.cn).

The sequence data were subject to similarity analysis using the BLAST program in NCBI (http://blast.ncbi.nlm.nih.gov/Blast.cgi). The DNASTAR program was used to analyze the open reading frame (ORF), and to predict the amino acid sequence. Signal peptide analysis was performed using SignalP 4.1 (http://www.cbs.dtu.dk/services/SignalP/). Subcellular localization was predicted using PSORT Prediction (http://psort.hgc.jp/form.html). Transmembrane protein structure was predicted by TMpred (http://www.ch.embnet.org/software/TMPRED_form.html) and TMHMM server v2.0 (http://www.cbs.dtu.dk/services/TMHMM/). Conservative domain was predicted by BLASTP in NCBI (http://blast.ncbi.nlm.nih.gov/Blast.cgi). A phylogenetic tree was constructed using the neighbor-joining (NJ) method in MEGA 5.05 (Tamura et al., 2011).

### Quantitative real-time PCR (qRT-PCR) analysis

qRT-PCR were performed to analyze the transcript accumulation of *LbKT1* and *LbSKOR* in leaves and roots. Primers used were listed in Table 1. qRT-PCR amplifications were performed with SYBR Premix Ex Taq™ II (Perfect Real Time; Takara Biotechnology Co., Ltd, China) according to the manufacturer's instructions. The amplifications were set at 20 μL reaction system including 10 μL SYBR Premix Ex Taq™ II, 0.8 μL forward and reverse primers (10 μmol/L), 7.4 μL nuclease-free water, and 1 μL cDNA (1:10 diluted with nuclease-free water). The reactions were performed on a Bio-Rad CFX96 real-time PCR instrument and the data were analyzed by using Bio-Rad CFX Manager software, version 1.1 (Bio-Rad, USA). A melting curve was recorded at the end of each run to detect primers generating non-specific PCR products (Ririe et al., 1997). A fragment of *L. barbarum* actin gene was used for normalization (Hu et al., 2017). The relative expression were calculated as 2^−ΔCT^ (ΔCT = CT^gene of interest^ − CT^actin^).

**Table 1:** **Primers used for gene clone and quantitative real-time PCR (qRT-PCR)**.

	**Primer name**	**GenBank accession**	**Primer sequence (5′-3′)**	**Product size (bp)**
Clone PCR	Primer1-for	LbKT1	ATGAATCAGATAACAACGGAAG	1,260
	Primer1-rev	fragment1	CCAGCAGAAGAACAACACAG	
	Primer2-for	LbKT1	TTAGTTGGCTGTCTCTACC	1,500
	Primer2-rev	fragment2	GTCTCCATTAGAACTCCT	
	Primer3-for	LbSKOR	TGATTACGAGGAGAGCAGA	1,002
	Primer3-rev	fragment1	TAATATACAAGAGCCCGACG	
	Primer4-for	LbSKOR	ATTAGCAGGCAAAGTGTT	1,520
	Primer4-rev	fragment2	AGCTGTTAGGCTCAAGAC	
qRT-PCR	LbKT1-for	KU523244	TTCCCAAGATCAACGGGTCATCGG	192
	LbKT1-rev		CTTATCACCATCCCGGATTCGAAG	
	LbSKOR-for	KU523245	CTTTTGATATGATTCTTGGTGCTT	181
	LbSKOR-rev		CTTTGATATTGTAATCGCAAGTGG	
	Actin-for	HQ415754	TCTACGAGGGTTACGCTTTG	126
	Actin-rev		TCCCGTTCAGCAGTGGTT	

### Statistical analysis

Analysis of variance (ANOVA) and Pearson's correlation analysis were performed by the program package Statistica (Version 9.1; StatSoft Inc., Tulsa, OK, USA). Fisher's LSD was performed at *P* = 0.05 in case of significant impact by factor.

## Result

### Plant growth, mycorrhizal colonization, and potassium content

Seventy days after transplantation, seedlings growth was recorded (Table 2). Inoculation of *R. irregularis* and application of potassium improved both shoot and root growth. Under each potassium condition, *R. irregularis* colonized *L. barbarum* seedlings had higher shoot and root weight. Compared with well-watered seedlings, drought stress reduced seedlings growth, and reduced the growth promotion by inoculation of *R. irregularis* and application of potassium. Colonization of *R. irregularis* obviously increased the root/shoot ratio under well-watered condition, while the potassium application increased the root/shoot ratio in non-mycorrhizal plants and decreased the root/shoot ratio in mycorrhizal plants. With 8 mM potassium application, the difference of root/shoot ratio between mycorrhizal and non-mycorrhizal plants was marginal. Under drought stress, influence of *R. irregularis* on root/shoot ratio was only obvious when 2 mM potassium was applied.

**Table 2:** **Biomass and mycorrhizal colonization of *L. barbarum* under different treatments**.

**Treatments**	**Shoot fresh weight (g)**	**Root fresh weight (g)**	**Root/shoot ratio**	**Colonization (%)**
DSNMK0	7.17 ± 0.15g	12.40 ± 0.36f	1.73 ± 0.01a	0
DSAMK0	7.43 ± 0.40g	12.67 ± 0.32f	1.71 ± 0.12a	61.60 ± 1.99
DSNMK1	10.07 ± 0.06f	13.40 ± 0.98ef	1.33 ± 0.10bcd	0
DSAMK1	11.70 ± 0.52de	13.47 ± 0.51ef	1.15 ± 0.06efg	62.47 ± 1.85
DSNMK2	11.40 ± 0.44e	14.10 ± 2.41e	1.24 ± 0.22def	0
DSAMK2	12.63 ± 0.35c	14.07 ± 0.15e	1.11 ± 0.03fg	68.71 ± 1.95
WWNMK0	11.37 ± 1.36e	16.13 ± 0.72cd	1.43 ± 0.11b	0
WWAMK0	13.60 ± 0.44b	14.53 ± 0.31e	1.07 ± 0.03g	58.42 ± 2.21
WWNMK1	12.50 ± 0.26cd	17.43 ± 0.29bc	1.40 ± 0.05bc	0
WWAMK1	14.27 ± 0.23ab	15.93 ± 0.21d	1.12 ± 0.03efg	61.06 ± 2.04
WWNMK2	13.83 ± 0.06ab	19.10 ± 0.10a	1.38 ± 0.01bcd	0
WWAMK2	14.53 ± 0.47a	18.37 ± 0.29ab	1.26 ± 0.04cde	64.98 ± 4.86
P_water_	s	s	s	s
P_AMF_	s	s	s	na
P_K_	s	s	s	s
P_water^*^AMF_	ns	s	s	na
P_water^*^K_	s	s	s	ns
P_AMF^*^K_	ns	ns	ns	na
P_water^*^AMF^*^K_	s	ns	ns	na

*Data was shown as mean ± SD; DS, drought stress; WW, well-water; NM, non-mycorrhizal; AM, inoculated with Rhizophagus irregularis; K0, applied with no KCl; K1, applied with 2 mM KCl; K2, applied with 8 mM KCl; s, significant; ns, not significant; na, not applicable; value with different letters indicated significant difference (LSD-test P = 0.05, n = 3)*.

No mycorrhizal colonization was observed in non-mycorrhizal seedlings. More than 58% roots of seedlings were colonized by *R. irregularis*, while the colonization was promoted by potassium application and drought stress. The highest colonization (68.71 ± 1.95%) was observed in the treatment under drought stress with 8 mM potassium application.

**Figure 1:**
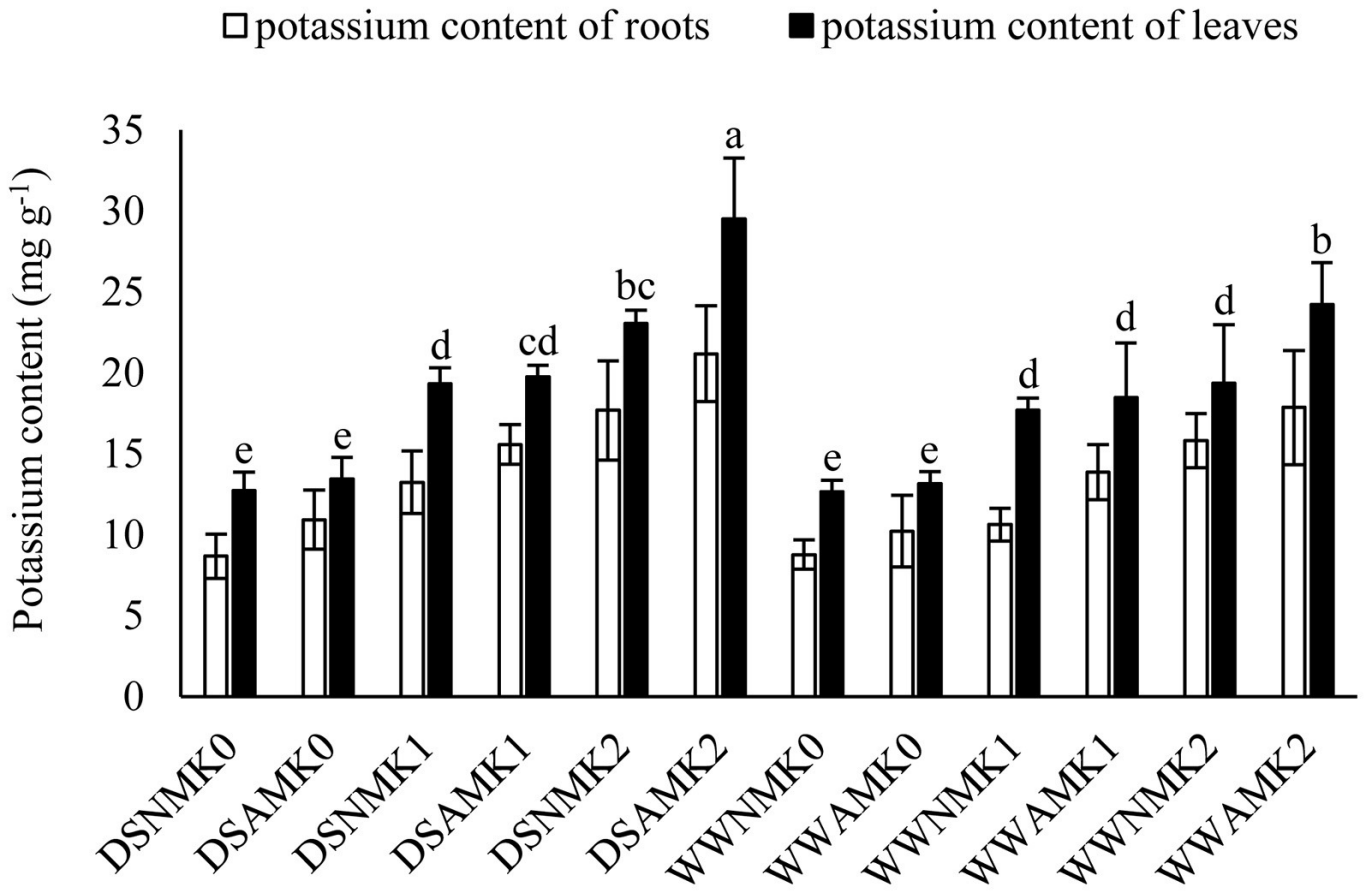
**Potassium content of *L. barbarum* roots and leaves under different treatments**. Different letters on columns indicated significant difference at *P* < 0.05 (LSD-test, *n* = 6). DS, drought stress; WW, well-water; NM, non-mycorrhizal; AM, inoculated with *Rhizophagus irregularis*; K0, applied with no KCl; K1, applied with 2 mM KCl; K2, applied with 8 mM KCl.

The potassium content in leaves was higher than that in roots (Figure 1). Both *R. irregularis* and potassium application improved potassium content in leaves and roots. Under drought stress, improvement of *R. irregularis* on potassium content in leaves and roots was only obvious when 8 mM potassium was applied.

### Analysis of two putative genes encoding shaker family potassium channels

Two novel full-length cDNA encoding Shaker family potassium channels were obtained, sequenced, designated *LbKT1* and *LbSKOR*, and deposited in GenBank (*LbKT1*, KU523244; *LbSKOR*, KU523245; Table 3). The open reading frame (ORF) for *LbKT1* was 2661 bp, encoded 886 amino acids, while the ORF for *LbSKOR* was 2448 bp, encoded 815 amino acids. The predicted molecular weight was 99.48 kDa for LbKT1 and 93.68 kDa for LbSKOR. Isoelectric point was 7.04 for LbKT1 and 6.61 for LbSKOR. Both LbKT1 and LbSKOR were predicted to locate on plasma membrane, and had no signal peptide.

**Table 3:** **Physiochemical properties and subcellular localization of LbKT1 and LbSKOR**.

**Designated gene name**	**GenBank accession**	**ORF length (bp)**	**Protein length (aa)**	**Molecular weight (kDa)**	**Isoelectric point**	**Subcellular location**	**Signal peptide**
*LbKT1*	KU523244	2661	886	99.48	7.04	Plasma membrane	NO
*LbSKOR*	KU523244	2448	815	93.68	6.61	Plasma membrane	NO

**Figure 2:**
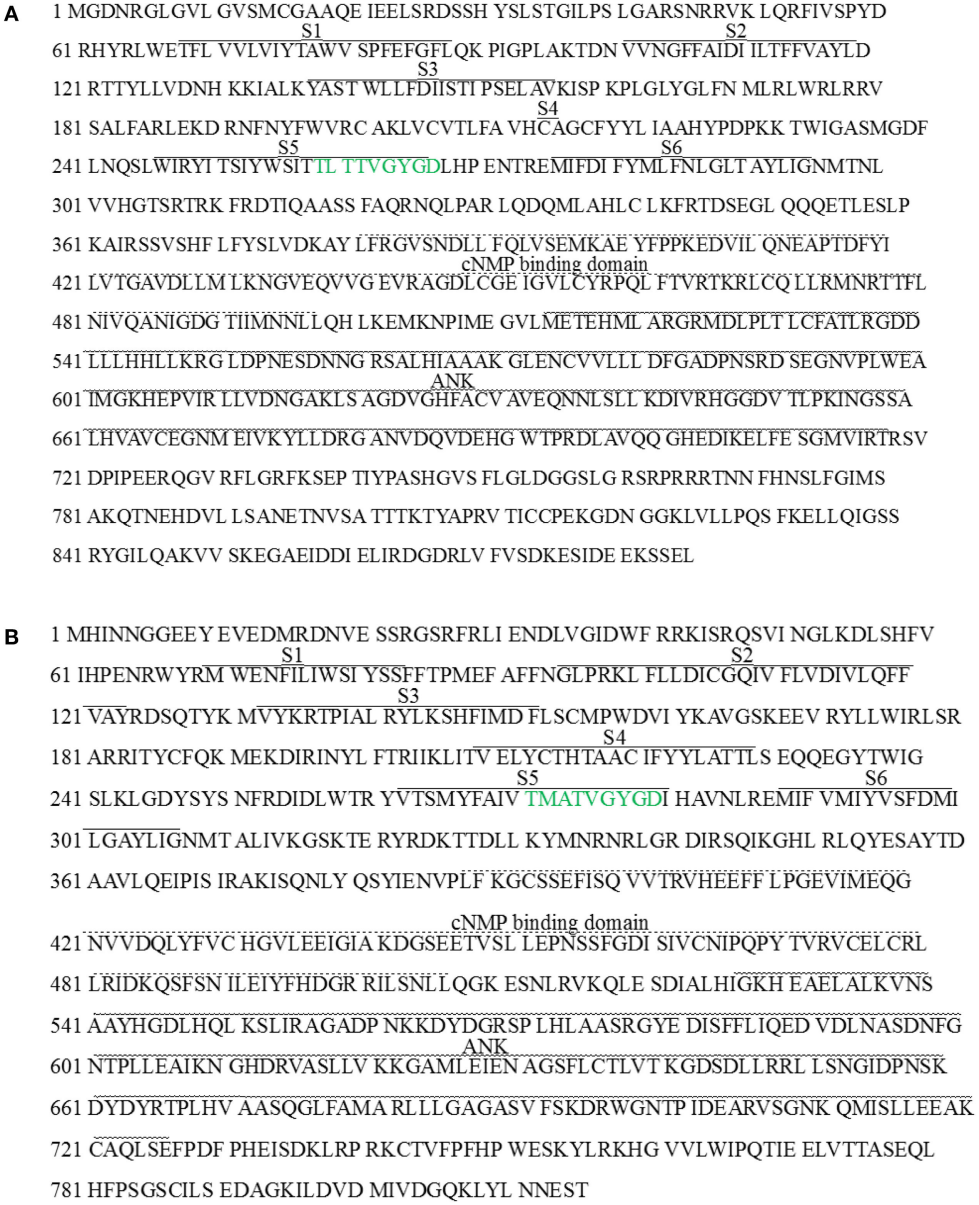
**Deduced amino acid sequence of *LbKT1* (A)** and *LbSKOR* **(B)**. Continuous line above the sequences indicated the putative trans-membrane domains (S1–S6), dotted line above the sequences indicated the putative cNMP binding domain, and curve above the sequences indicated the putative ankyrin repeats domain.

**Figure 3:**
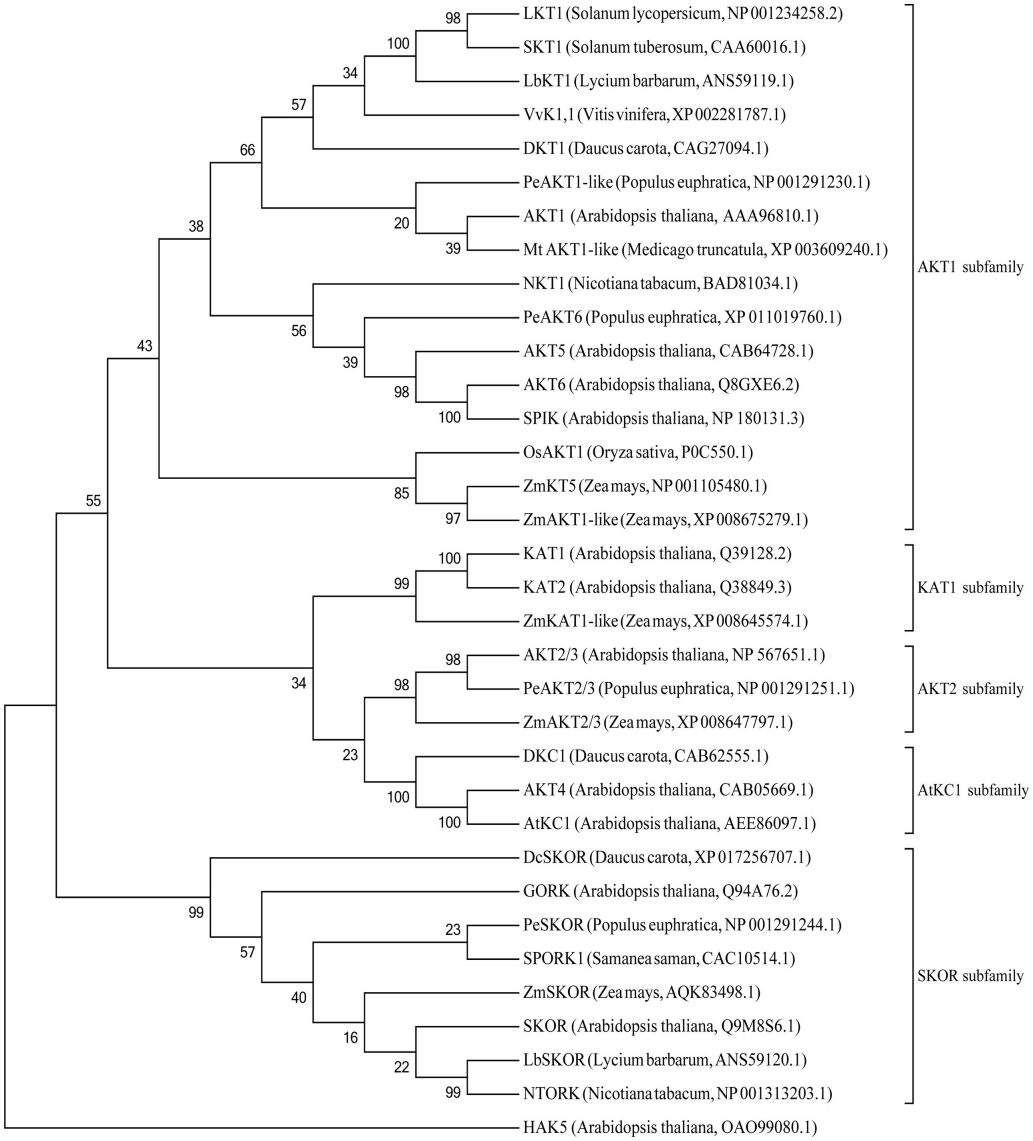
**Phylogenetic tree of plant potassium channels**. The tree was constructed with MEGA5 using the neighbor-joining (NJ) method. Values indicated at the nodes were bootstrap values based on 1,000 replicates.

Based on the deduced amino acid sequence, analysis of protein conserved domain indicated that both LbKT1 and LbSKOR had six transmembrane domains (S1–S6), one cNMP binding domain, and one ankyrin repeats domain (Figure 2). Between S5 and S6, there was one P-loop which have the signature sequence of potassium channel (TxxTxGYGD, green in Figure 2). Based the constructed phylogenetic tree (Figure 3), LbKT1 belonged to the inward-rectifying AKT1 subfamily, and LbSKOR was a member of the outward-rectifying SKOR subfamily.

### Expression of *LbKT1* and *LbSKOR* in leaves and roots

**Figure 4:**
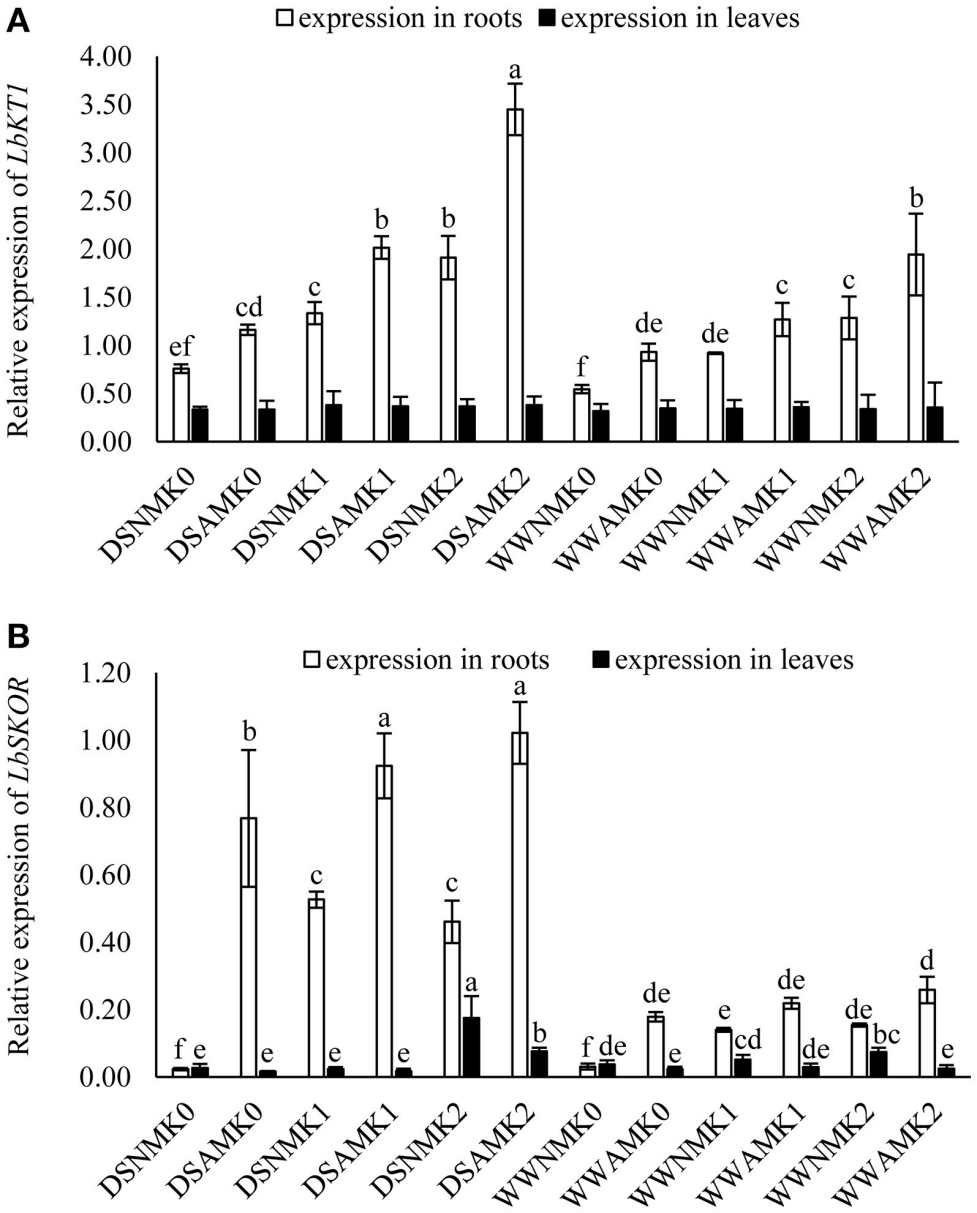
**Relative expression of *LbKT1* (A)** and *LbSKOR* **(B)** in roots and leaves of *Lycium barbarum*. Different letters on columns indicated significant difference at *P* < 0.05 (LSD-test, *n* = 6). DS, drought stress; WW, well-water; NM, non-mycorrhizal; AM, inoculated with *Rhizophagus irregularis*; K0, applied with no KCl; K1, applied with 2 mM KCl; K2, applied with 8 mM KCl.

In roots, inoculation of *R. irregularis*, application of potassium, and water stress improved the expression of *LbKT1* and *LbSKOR*. Under the same potassium content in soil, mycorrhizal plants had higher *LbKT1* and *LbSKOR* expression in roots than that in non-mycorrhizal plants. When 2 and 8 mM KCl were applied, the *LbKT1* and *LbSKOR* expression in roots under drought stress was significant higher than that under well-watered condition (Figure 4). In leaves, inoculation of *R. irregularis*, application of potassium, and water stress did not influence the expression of *LbKT1*. When 0 and 2 mM potassium was applied, *R. irregularis* and drought stress did not influence the expression of *LbSKOR* in leaves. When 8 mM potassium was applied, drought stress increased the *LbSKOR* expression while *R. irregularis* decreased its expression in leaves (Figure 4).

### Correlation analysis

The expression of *LbKT1* in roots was highly correlated with the potassium content of roots and leaves (*P* < 0.001), and the correlation coefficient was higher than that of *LbSKOR* (*P* < 0.01; Table 4). Both the expression of *LbKT1* and *LbSKOR* in roots was highly correlated with the colonization (*P* < 0.01). The expression of *LbSKOR* in leaves was correlated with the potassium content of leaves (*P* < 0.05). The mycorrhizal colonization was correlated with the potassium content of roots (*P* < 0.05).

**Table 4:** **Correlation coefficients among potassium content, gene expression, and mycorrhizal colonization**.

	**Expression of *LbKT1* in roots**	**Expression of *LbKT1* in leaves**	**Expression of *LbSKOR* in roots**	**Expression of *LbSKOR* in leaves**	**Colonization**
Potassium content of roots	0.82^***^	−0.11	0.50^**^	0.32	0.33^*^
Potassium content of leaves	0.84^***^	−0.05	0.50^**^	0.39^*^	0.27
colonization	0.46^**^	0.09	0.54^***^	−0.30	1

* *Indicated significance of correlation coefficient at P < 0.05*.

** *Indicated significance of correlation coefficient at P < 0.01*.

*** *Indicated significance of correlation coefficient at P < 0.001*.

## Discussion

Establishment of symbiosis was the start point of AM fungi to promote plant mineral and water absorption and resistance/tolerance against biotic and abiotic stresses (Smith and Read, 2008). More than 58% roots of *L. barbarum* seedlings were colonized by *R. irregularis* 70 days after inoculation in current study. This was consistent with previous survey that *L. barbarum* was capable of forming AM (Zhang et al., 2010). In the study of El-Mesbahi et al. (2012), potassium application increased only the AM fungi colonized root length, but not the colonization rate. Similar result was obtained in current study, application of potassium increased both the colonization rate and the seedling root growth, which resulted also in the increased AM fungi colonized root length.

In current study, the seedlings of *L. barbarum* was fertilized with Hoagland solution contained full strength phosphate and nitrogen. Due to the extra application of potassium, the balance among potassium, phosphate, and nitrogen was modified, and might increase the demand of *L. barbarum* for more phosphate and nitrogen. The relative shortage of phosphate and nitrogen in the environment, instead of inner nutrient concentration, might be sensed by plant and would explain the increased colonization rate of *R. irregularis* (Bonneau et al., 2013). Drought stress limited the diffusion of minerals in soil, and further aggregated the imbalance among potassium, phosphate and nitrogen. This might be the reason of the highest colonization rate of *R. irregularis* in the treatment under drought stress with 8 mM potassium application (Table 2).

Plant growth is the most obvious trait for the beneficial effect of AM fungi under abiotic stress (Evelin et al., 2009; Ruiz-Lozano et al., 2012a,b). In current study, inoculation of *R. irregularis* improved the growth of *L. barbarum* seedlings, which was consistent with previous studies that focused on the interactions between AM fungi and the solanaceous plant species (Yao et al., 2002; Kaya et al., 2009; Boldt et al., 2011). This might due to the *R. irregularis* improved mineral absorption (Smith et al., 2003) and the improved root hydraulic conductivity for water absorption (El-Mesbahi et al., 2012). Under well-watered condition, inoculation of *R. irregularis* obviously reduced the root/shoot ratio (Table 2). This was consistent with previous studies that the mycorrhizal plants had a lower root/shoot ratio than their non-mycorrhizal counterparts, and this might due be to the reliance of plants on AM fungal mycelium (Zhang et al., 2016). Under drought stress, the effect of *R. irregularis* on root/shoot ratio was only obvious when extra potassium was applied. This might attribute to the nutrient translocation strategy that plant cope with water stress (Chaves et al., 2003; Jaramillo et al., 2013). Facing drought stress, plants invested more photosynthates into root growth to absorb water and mineral nutrients. As plant growth was correlated with potassium uptake (Rubio et al., 2010; Alemán et al., 2011), potassium application improved root growth, which satisfied the demand of water and mineral nutrients absorption. Then, the balance between shoot and root growth changed, and the influence of *R. irregularis* emerged (Egilla et al., 2001).

Under well-watered condition, the potassium application decreased root/shoot ratio in non-mycorrhizal plants and increased root/shoot ratio in mycorrhizal plants (Table 2). This implied that potassium application increased the reliance of plant on root and decreased the reliance of plant on AM fungal mycelium. The changed reliance might due to the potassium stimulated systemic lateral root growth improved water and mineral nutrients absorption (Drew, 1975). Another possibility might be the nutrient foraging strategy of *L. barbarum*, which relied on root growth rather than AM fungal mycelium (Chen et al., 2016). Combined with the higher efficiency of root hair in nutrient absorption (Brown et al., 2013) and substitution of AM fungal mycelium on root hair (Jakobsen et al., 2005), this might be another potential explanation for the changed reliance. Although the root/shoot ratio in non-mycorrhizal plants was lowered, it was still higher than that in mycorrhizal plants. This implied that AM fungal mycelium was better in carbon cost than root hair in nutrient uptake (Orfanoudakis et al., 2010), and had further access beyond nutrient depletion zone around root (Jakobsen et al., 2005).

Potassium involves in different metabolism processes (Wang and Wu, 2013; Garcia and Zimmermann, 2014). Inoculation of *R. irregularis* increased the potassium content both in leaves and roots (Figure 1). This was consistent with some studies (Porras-Soriano et al., 2009; Baslam et al., 2013; Estrada et al., 2013), while El-Mesbahi et al. (2012) found a reduced potassium content in mycorrhizal maize shoot. This might due to the different plants and AM fungi combinations, and condition for plant cultivation. Although George et al. (1992) indicated that the plant potassium uptake was increased when the AM fungus had access to the extra potassium in another compartment, the direct evidence that AM fungi transport potassium to plant was still rare. Using particle-induced X-ray emission analysis, a strong accumulation of potassium was documented in different parts of AM fungus (Pallon et al., 2007; Olsson et al., 2008, 2011), and the mycorrhizal root section (Scheloske et al., 2004). All these observations suggested that AM fungi are capable of transporting potassium to plants. The positive correlated potassium and phosphate content in plants (Olsson et al., 2011), and the suggested role of main counter-ion for electrochemical polyphosphate stabilization (Orlovich and Ashford, 1993; Kikuchi et al., 2014) made potassium an indispensable mineral element for the mycorrhizal pathway of phosphate transporting. Under drought stress, plant required more potassium to improve its tolerance/resistance via optimizing leaf water content, photosynthesis, water-use efficiency, and antioxidative enzymes activity (Egilla et al., 2001, 2005; Soleimanzadeh et al., 2010). In current study, drought stress increased potassium concentration in both roots and leaves (Figure 1). This was consistent with the study of Egilla et al. (2001). More studies are still needed to illustrate the uptake of potassium via AM fungi and its specific contribution to the improved plant drought tolerance/resistance.

Plant potassium channels are multimeric proteins. The channels from Shaker family had six transmembrane domains, one pore (P) domain between transmembrane 5 and 6, while the P domain contained signature motif TxxTxGYGD (Lebaudy et al., 2007). In current study, two novel full-length cDNA were obtained and designated *LbKT1* and *LbSKOR* due to their sequences and domains similarity with genes encoding Shaker family potassium channels (Figures 2, 3).

In Arabidopsis, AKT1 had been localized to epidermis, cortex and endodermis (Cao et al., 1995; Lagarde et al., 1996), while its homolog LKT1 from tomato and OsAKT1 from rice was localized to root hair, epidermis and endodermis (Hartje et al., 2000; Golldack et al., 2003). In charge of potassium uptake from soil, AKT1 was reported respond to a wide range of potassium concentrations (10 μM–10 mM; Wang and Wu, 2013). In current study, expression of *LbKT1* in root was increased by the potassium application (Figure 4), and positively correlated with the potassium content in roots and leaves (Table 4). This suggested that LbKT1, similar to AKT1, LKT1 and OsAKT1, played the potassium uptake role in *L. barbarum*. Inoculation of *R. irregularis* increased the *LbKT1* expression in roots, which paralleled with the AM fungus improve potassium content, indicated that *R. irregularis* provided extra potassium in *L. barbarum* roots. The upregulation of *LbKT1* in mycorrhizal roots needs further experiments to pinpoint the specific role of AM fungi. Drought stress in current study also increased the expression of *LbKT1*. This might be the respond of *L. barbarum* to drought stress by increasing potassium uptake to lower cellular water potential (Ahmad et al., 2016) and regulate stomatal conductance (Nieves-Cordones et al., 2012). Expression of *LbKT1* was also detected in *L. barbarum* leaves. This was consistent with previous studies that *AKT1* was expressed in Arabidopsis mesophyll cell (Dennison et al., 2001), while *OsAKT1* in rice was detected in mesophyll cell and cells neighboring the metaxylem vessels (Golldack et al., 2003). Drought, inoculation of *R. irregularis*, and potassium application in current study did not affect the expression of *LbKT1* in leaves. This was contradicted with the study of *VvK1.1* in Grapevine (Cuéllar et al., 2010), in which drought increased the expression of *VvK1.1* in leaves and decreased its expression in roots. These contradicted expression results might due to the different strategies that plants cope with drought stress.

SKOR is the potassium channel plays a key role in root-shoot potassium translocation by secretion of potassium into xylem sap (Gaymard et al., 1998). In current study, expression of *LbSKOR* in roots was positively correlated with the potassium content in roots and leaves, and the colonization of *R. irregularis* (Table 4). This correlation suggested the potassium translocation function of LbSKOR in *L. barbarum* and the *LbSKOR* expression was increased by the AM fungal colonization, which was consistent with the study of Estrada et al. (2013). Upregulation of *LbSKOR* expression in roots by *R. irregularis*, especially under drought stress (Figure 4), suggested an extra potassium supplement by AM fungus favored the requirement of plant (Pilot et al., 2003), and confirmed the role of SKOR in potassium homeostasis under drought stress (Hu et al., 2016). Expression of *LbSKOR* was also detected in leaves, and positively correlated with the potassium content in leaves (Table 4). Similar to its homologs *SPORK1* in *Samanea saman* (Moshelion et al., 2002) and *NTORK* in tobacco (Sano et al., 2007), *LbSKOR* might in charge of potassium permeability in *L. barbarum* leaves, while its specific localization and function needs further study.

In conclusion, our results indicated that the potassium status of *L. barbarum* was modulated by *R. irregularis*, soil water status, and potassium content in soil. The putative genes *LbKT1* and *LbSKOR* belonged to the Shaker family were in charge of potassium uptake and translocation, and differently regulated by AM fungus, water status and potassium availability. The improved *L. barbarum* potassium content by the colonization of *R. irregularis* suggested a mycorrhizal potassium transport pathway (Garcia and Zimmermann, 2014). However, the transport of potassium in the interface between plant and AM fungus remains elusive. Since AM fungus specifically induced genes encoding potassium transporter and channel were discovered (Guether et al., 2009; personal transcriptome data), future studies illustrate the function and location of these genes could reveal the contribution of AM fungus in plant potassium nutrient.

## Author contributions

HZ, WH, and MT designed this experiment. HZ, SW, and LX carried out the experiment, and gathered the data. HZ and MT interpreted the results, drafted and revised the manuscript. HZ and MT final proved the manuscript, and agreed to be accountable for all aspects of the work in ensuring that questions related to the accuracy or integrity of any part of the work are appropriately investigated and resolved.

### Conflict of interest statement

The authors declare that the research was conducted in the absence of any commercial or financial relationships that could be construed as a potential conflict of interest.
